# Application of an innovative isoniazid-loaded biomimetic mineralized bone collagen scaffold in bone defect treatment

**DOI:** 10.1039/d5ra03800f

**Published:** 2025-08-13

**Authors:** Qian Wang, Xu Fang, Aihua Feng, Jun-feng Dong

**Affiliations:** a Department of Spine Surgery, Shanghai East Hospital, Tongji University School of Medicine Shanghai China; b Department of Spinal Surgery, The First Dongguan Affiliated Hospital, Guangdong Medical University Guangdong China doctor_d0346@163.com; c Department of Obstetrics Department, Dongguan Southeast Central Hospital Guangdong China

## Abstract

Bone defects, arising from various causes such as trauma, infection, tumor, surgical debridement, and congenital diseases, pose challenges to the natural self-healing process of bone tissue. Large-scale bone defects may lead to non-healing of the bone, and they need to be repaired through surgery using transplant materials. While graft materials serve as scaffolds for cell attachment and growth, infectious bone defects, like tuberculosis of the bone and joint, require anti-infective therapy concurrent with defect repair. This study aimed to construct a nano-scale drug-loaded mineralized collagen scaffold and assess its physicochemical, anti-infective, and osteogenic properties. By using isoniazid, hydroxyapatite and type I collagen, we achieved assembly through biomimetic mineralization principle and prepared a drug-loaded mineralized collagen scaffold. The scaffolds' surface morphology and composition were analyzed *via* field emission scanning electron microscopy (SEM) and X-ray diffraction (XRD). After sterilization, the scaffolds were co-cultured with tuberculosis bacteria to evaluate the inhibition of *Mycobacterium tuberculosis* growth. Additionally, the scaffolds were implanted into mice to assess degradation and drug sustained release. In a critical bone defect model in SD rats, osteogenesis was detected by Micro-CT, and biocompatibility was evaluated using HE staining of vital organs. The drug loading rate and entrapment efficiency of drug-loaded scaffolds were (6.25 ± 0.48)% and (54 ± 2.34)%, respectively. In co-culture with tuberculosis bacteria, the drug-loaded group showed a negative result in the BACTEC MIGT 960 detection system after continuous observation for more than 8 weeks, contrasting with positive results in the blank and non-drug-loaded groups. At the 8th week, acid-fast staining (AFS), auramine o staining, Micro-CT, and HE staining confirmed the drug-loaded scaffold's antibacterial properties, sustained-release capabilities, biocompatibility, and osteogenesis. Our findings demonstrate that drug-loaded biomimetic mineralized collagen scaffolds exhibit sustained-release properties, biodegradability, antibacterial efficacy, biocompatibility, and osteogenic potential. This novel drug-loaded collagen scaffold holds significant promise for the effective repair of infected bone defects.

## Abstract 2

We have developed a novel type of mineralized collagen scaffold loaded with isoniazid, which exhibits excellent drug release properties, degradability, anti-tuberculosis performance, biocompatibility and osteogenicity.

## Graphical abstract

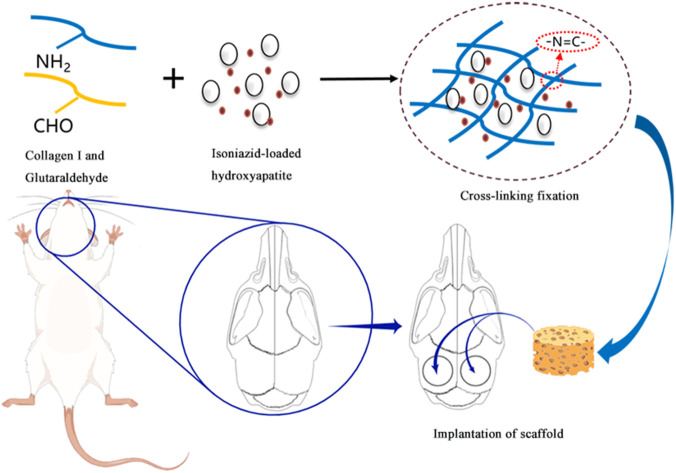

## 1 Introduction

Infectious bone defects are caused by the infection of bone tissue by pathogens. They are mainly characterized by osteomyelitis and bone loss. Tuberculous bone defect is a common type of infectious bone defect. Osteoarticular tuberculosis, constituting 10–34% of extrapulmonary tuberculosis, is particularly prevalent, characterized by a high disability rate and protracted healing processes.^1,2^ Afflicting weight-bearing areas like vertebral bodies, knees, and hips, bone and joint tuberculosis lead to destructive lesions, sinus and abscess formation, bone degradation, pathological fractures, local deformities, and, in severe cases, may even cause paraplegia, significantly impacting individuals' health and quality of life.^3,4^ Addressing bone and joint tuberculosis remains a formidable challenge in the medical field, with current treatments encompassing drug therapy and surgery. Drug therapy primarily involves traditional intramuscular, oral, or intravenous tuberculosis management, aiming to eradicate the tuberculosis bacteria through systemic circulation. However, prolonged systemic drug administration poses risks of drug accumulation in organs, resulting in severe liver and kidney damage, peripheral neuropathy, and gastrointestinal reactions. Moreover, ensuring effective drug distribution in the lesion area without inducing toxic effects on other organs remains challenging.^5,6^ Surgical intervention, particularly the use of bone repair materials, is a key approach to treating tuberculosis-induced bone defects. Commonly employed materials include autologous bone, allogeneic bone, titanium mesh, and calcium phosphate ceramics.^7,8^ While autogenous bone remains the gold standard due to its excellent osteoinductive properties, its limited availability and potential complications, such as deformities and infections at the extraction site, necessitate alternative solutions. Allogeneic bone exhibits good biocompatibility but carries a risk of infection. Titanium mesh, while effective for early healing, faces challenges in degradation.

**Fig. 1:**
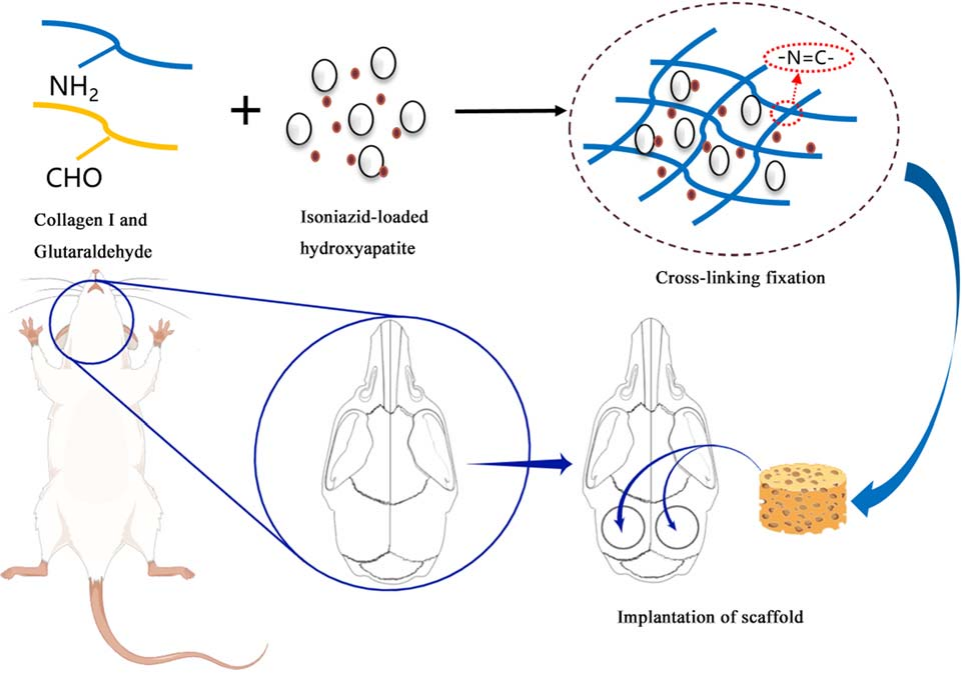
Schematic of the experimental process (generated using Figdraw).

In response to these challenges, a novel bone tissue engineering scaffold with tuberculosis management delivery functionality has been designed based on drug delivery system theory and tissue engineering principles. Hydroxyapatite (HA) and collagen I (Col I), as primary components of natural bone, are assembled through hydroxyapatite steps.^9^ This study aims to utilize Col I and HA as raw materials, based on the principle of biomimetic mineralization, and through molecular co-precipitation technology, to construct a novel drug-loaded mineralized collagen scaffold (Fig. 1). Evaluate the potential of this scaffold in treating infectious bone defects. This study covers aspects such as degradation, drug release, anti-infection ability, biological safety, and osteogenic properties.

## 2 Materials and methods

### 2.1 Experimental materials

Isoniazid was procured from shanghai-based McLin biochemical. Collagen type was obtained from China National Pharmaceutical Chemical Reagent Co., Ltd. Phosphate-buffered saline (PBS) was acquired from Hyclone in the USA. Kunming rats and SD rats were sourced from Hubei Entes Biotechnology Co., Ltd. The bone drill was obtained from Changsha Tiantian Dental Equipment Co., Ltd.

### 2.2 Preparation of isoniazid-loaded collagen scaffolds

(1) Isoniazid-loaded hydroxyapatite preparation: A 100 ml solution of 0.125 mol L^−1^ calcium nitrate in anhydrous ethanol was prepared. The solution was heated to 60 °C, followed by the addition of isoniazid. The temperature was then cooled to 37 °C to induce isoniazid supersaturation. Subsequently, 10 ml of 0.75 mol L^−1^ ammonium dihydrogen phosphate solution was added dropwise, accompanied by continuous stirring. The pH of the solution was adjusted to 9–11 using a moderate quantity of sodium hydroxide solution. After 4 h of stirring, the mixture was incubated overnight in a 37 °C thermostatic bath. The supernatant was removed, leaving a layer of precipitate, which was washed three times with anhydrous ethanol. The resulting material was dried in a −80 °C freeze-dryer for 24 h, yielding isoniazid-loaded hydroxyapatite. (2) Collagen solution preparation: Gelatin (1.5 g) was dissolved in 100 ml of deionized water through heating, resulting in a 15% collagen solution. (3) Uniform dispersion of 0.3 g isoniazid-containing hydroxylapatite nanoparticles in the collagen solution. (4) Injection of the hydroxylapatite collagen mixture into a suitably sized mold, followed by placement in an ice-water bath for complete solidification of collagen into a hydrogel. (5) Fixation of hydrogels with pre-cooled glutaraldehyde for 15 min, followed by drying in a −80 °C freeze-dryer for 24 h to obtain drug-loaded scaffolds. Isoniazid-free mineralized collagen scaffolds were similarly prepared.

### 2.3 Characterization and physicochemical properties of the scaffolds

(1) The scaffold's microstructure was examined through field emission scanning electron microscopy. (2) Phase analysis of the scaffold was conducted using an X-ray diffractometer. (3) Determination of drug loading and entrapment efficiency of the scaffold involved the following method: A specific quantity of isoniazid was weighed and dissolved in a predetermined volume of PBS solution, resulting in an isoniazid mother liquor of 200 μg ml^−1^. Subsequently, 1.0, 2.0, 3.0, 4.0, 5.0, and 6.0 ml of the mother liquor were separately transferred into volumetric flasks and adjusted to 100 ml with PBS. The absorbance of these six solutions at 263 nm was measured using a UV photometer, and standard curves were generated through regression analysis. The scaffolds were weighed appropriately, thoroughly ground in a mortar, and dissolved in PBS. The mixture was stirred at a constant speed for 24 h, followed by centrifugation to remove sediment. The supernatant was retained, and PBS was added to reach a total volume of 100 ml. The absorbance of the solution was measured with a UV photometer. Utilizing the regression curve, drug concentration was calculated, allowing for the subsequent determination of drug loading rate (DLR) and entrapment efficiency (EE) using the formulas [DLR = (The drug content in the solution)/(scaffold quality); EE = (The drug content in the solution)/the average amount of the drug used].

### 2.4 *In vitro* anti-tuberculous activity of scaffold

A bacterial suspension of 1.5 × 10^4^ cfu ml^−1^ was prepared using PBS, and 1 ml of this suspension was introduced into each standard MGIT liquid culture tube, followed by incubation at 37 °C. The experiment encompassed three groups: Experimental Group, Control Group, and Blank Group, each consisting of 10 subjects. Isoniazid-loaded scaffolds were introduced into the culture tubes of the Experimental Group, isoniazid-free scaffolds in the Control Group, and no materials were added to the culture tubes of the Blank Group. Indicators and test methods employed were as follows:

(1) BACTEC MGIT 960 System Detection: The MGIT 960 system automatically assessed the culture results of *Mycobacterium tuberculosis* in the liquid MGIT medium across the three experimental groups.

(2) Auramine O Staining: At the 8th week, smears from the culture tubes were stained using the Auramine O staining kit. Subsequently, the culture tubes were examined using a fluorescence microscope.

(3) Acid-Fast Stain: At the 8th week, smears from the culture tubes were stained using the AFS kit.

### 2.5 Drug sustained release and scaffold degradation *in vivo*

We selected 36 healthy Kunming mice, each weighing between 18 and 20 g, and prepared 36 scaffolds of uniform size, each weighing 10 mg (*W*_0_). Anesthesia was induced in the mice through intraperitoneal injection of 1% sodium pentobarbital. Following this, an incision was made in the dorsal skin of the mice, creating a subcutaneous pouch into which the scaffold was implanted. Subsequently, 3 mice were treated every week over a period of 1 to 12 weeks. The scaffolds were then extracted, and any biofilm on the scaffold surface was removed. The remaining scaffolds underwent freeze-drying, and their weight (*W*_r_) was recorded. The cumulative weight loss rate was calculated and expressed as WLR (%). The scaffolds were crushed, combined with 20 ml of PBS, stirred at a constant speed for 24 h, centrifuged to remove sediment, and then PBS was added to achieve a total volume of 100 ml. The UV photometer was employed to measure the solution's absorbance, and the concentration of the residual drug in the solution (*C*_1_) was calculated. The initial drug concentration of a 10 mg scaffold completely dissolved in water was denoted as *C*_0_. The drug release fraction was documented as CRP (%). Formula: WLR(%) = (*W*_0_ − *W*_r_)/*W*_0_ × 100%; CRP (%) = (*C*_0_ − *C*_1_)/*C*_0_ × 100%.

### 2.6 Bone formation and biosafety of the scaffold *in vivo*

**Fig. 2:**
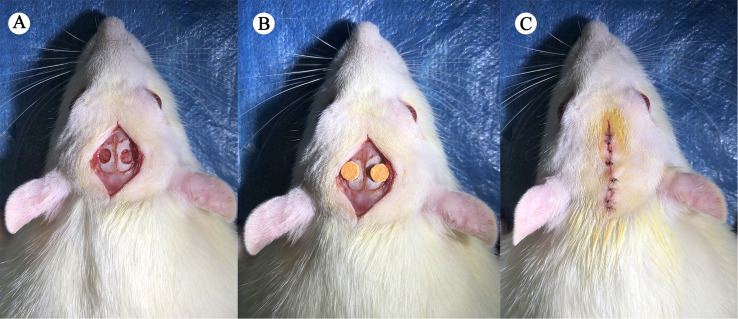
Modeling procedure. (A) Creation of two 5 mm bone defects. (B) Scaffold implantation in the experimental group. (C) Wound closure through suturing.

Prepare the scaffold into a cylindrical shape with a diameter of 5 mm and a thickness of 0.5 mm for future use. Some literature indicates that when the diameter of the bone defect is too small, the skull has a strong self-repairing ability, and 5 mm is a critical size. Therefore, we chose to create two 5 millimeter-sized defects on the skull of SD rats.^10^ Take 300–350 g of SD rats, and randomly divide them into 4 groups (experimental group 1, experimental group 2, control group 1, control group 2), with 8 rats in each group. Anesthesia using sodium pentobarbital was administered to the SD rats, followed by hair removal from their heads and disinfection with iodophor. An incision was made along the cranial suture using a scalpel, exposing the skull through blunt separation with a surgical forceps. Subsequently, 5 mm round holes were drilled on both sides of the middle cranial suture, and the scaffolds were implanted and sutured (Fig. 2). In the control group, the skull was drilled and sutured without scaffold implantation. Throughout the procedure, utmost care was taken to avoid damaging the dura mater and minimize injury to blood vessels during soft tissue separation. Two days post-operation, rats received carlofen analgesia and penicillin to prevent infection. Osteogenesis in Experiment Group 1 and Control Group 1 was assessed at 6 weeks post-operation, while that of Experiment Group 2 and Control Group 2 was evaluated at 12 weeks post-operation using Micro-CT for bone formation detection. After 12 weeks of modeling, hearts, livers, lungs, and kidneys from both the experimental and control groups were collected and processed into paraffin sections for HE staining. Pathological changes in vital organs were scrutinized to assess the biological safety of the scaffold.

### 2.7 Statistical analysis

The analysis and generation of necessary charts were conducted using GraphPad Prism 8.0.2 statistical software. Measurement data are presented as mean ± standard deviation, and the comparison between the two groups was executed through an independent sample *t*-test. A *p*-value less than 0.05 indicated a statistically significant difference. Image manipulation was carried out using Photoshop CS6 and Figdraw.

## 3 Results

### 3.1 Appearance and physicochemical properties of the scaffold

**Fig. 3:**
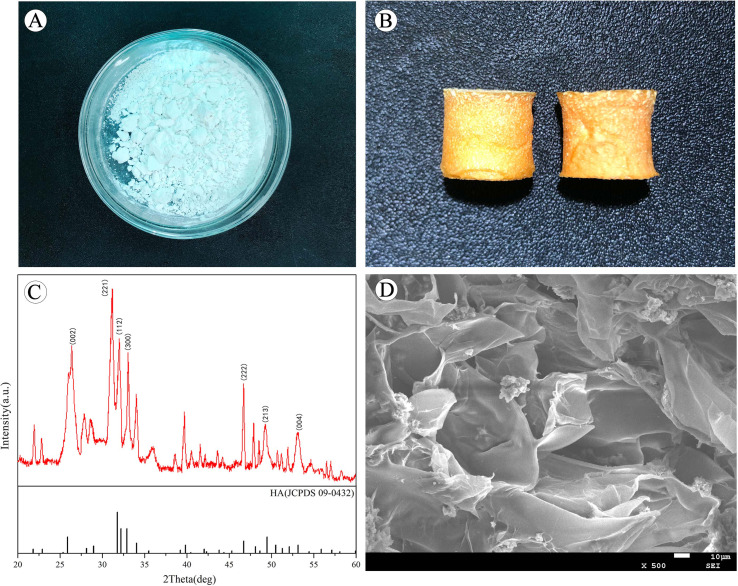
Scaffold appearance and characterization. (A): Calcium phosphosphate nanoparticles loaded with isoniazid. (B): Appearance of isoniazid-loaded biomimetic collagen scaffold. (C): X-ray diffraction patterns of isoniazid-loaded scaffolds. (D): ESEM diffraction patterns of isoniazid-loaded scaffolds.

Isoniazid-loaded hydroxyapatite appeared as a white powder (Fig. 3A), while scaffolds containing isoniazid exhibited a yellow, porous sponge-like structure (Fig. 3B). X-ray diffraction (XRD) analysis of the scaffolds revealed characteristic diffraction peaks on Crystal Faces (002), (211), (112), (300), (222), (004), (004). The diffraction peak consistency with the nano-hydroxyapatite (nHA) standard card (JCPDS 73-0432) indicated that the scaffold's phase remained unchanged during preparation, with the main inorganic component being nHA. Scanning electron microscope images (Fig. 3D) depicted a porous structure with high porosity in the scaffolds. Isoniazid-loaded hydroxyapatite was co-deposited with type I collagen at a molecular weight ratio of 1 : 5. The drug loading and encapsulation rate were (6.25 ± 0.48)% and (54 ± 2.34)%, respectively. The results affirm the stability of the scaffold's phase and display its porous nature, crucial for potential applications in drug delivery systems.

### 3.2 Anti-tuberculosis performance of the scaffold *in vitro*

#### 3.2.1 BACTEC MIGT 960 system detection results

Upon reaching a specific concentration of M. Tuberculosis in the MGIT culture tube, the BACTEC MIGT 960 system automatically signaled a positive status. In the blank group, positive cells first appeared on the 7th day and were universally present by the 10th day, with an average detection time of 9.11 ± 1.36 days. In the scaffold-only group without drug loading, positive expression commenced on the 10th day and was uniformly observed by the 16th day, resulting in an average detection time of 12.83 ± 2.9 days. In contrast, the drug-loaded scaffold group underwent continuous monitoring for 8 weeks, revealing no positive results throughout the observation period.

#### 3.2.2 Auramine “O” staining results

**Fig. 4:**
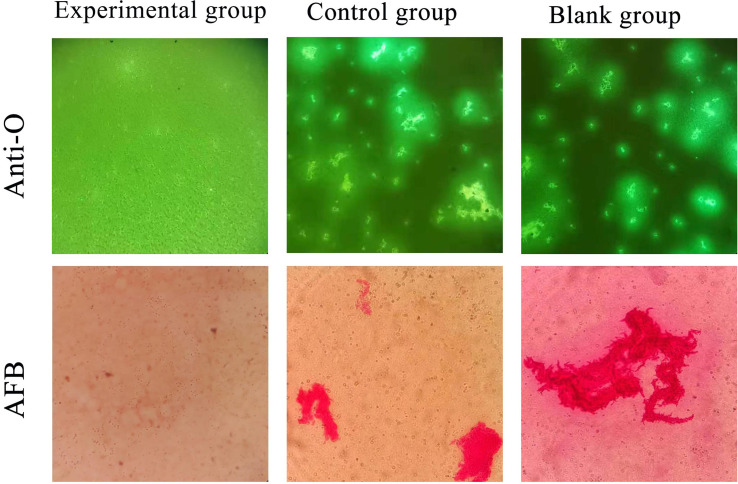
Auramine “O” and acid-fast staining (AFB).

In the eighth week, three randomly chosen tubes from each of the three groups underwent smearing and staining with auramine “O” (AuramineO). Fig. 4 illustrates that, at this juncture, *Mycobacterium tuberculosis* was notably absent in the experimental group as evidenced by auramine “O” staining. In contrast, distinct clusters of *Mycobacterium tuberculosis* were observed in both the control and blank groups. This observation signifies the efficacy of isoniazid-loaded scaffolds in the experimental group, demonstrating their capability to effectively resist *Mycobacterium tuberculosis*.

#### 3.2.3 Acid fast staining results

In the eighth week, three culture tubes from each group were randomly selected and subjected to AFS. Significant differences in AFS were observed among the three groups. As illustrated in Fig. 4 at the 8 weeks mark, *Mycobacterium tuberculosis* was not detected in the experimental group following acid-fast staining. In contrast, the control group and the blank group exhibited conspicuous red tuberculosis bacilli.

### 3.3 *In vivo* drug release and degradation

**Fig. 5:**
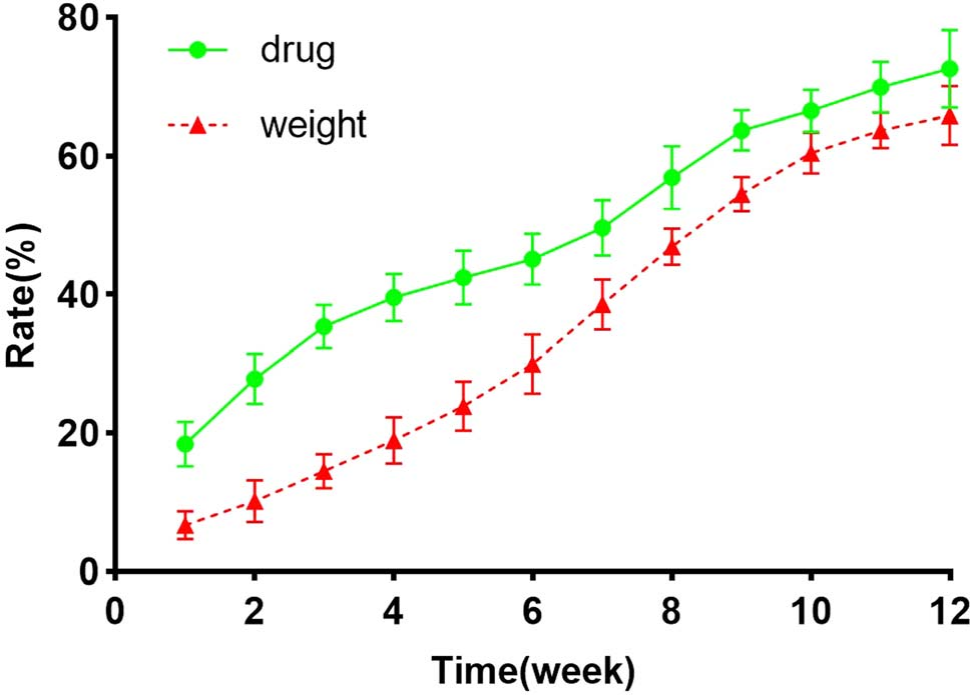
Cumulative weight loss of scaffold and sustained drug release.

Utilizing the correlation between isoniazid concentration and absorbance at 265 nm in PBS, the regression equation is expressed as follows: *A* = 0.001557 × *C* − 0.001400 (*R*^2^ = 0.9897, *n* = 6). Employing this equation, the corresponding drug concentrations were calculated, enabling the generation of cumulative drug release curves and scaffold weight loss rates, as depicted in Fig. 5. During the initial week, a substantial amount of drug (18.45 ± 3.21)% was rapidly released from the scaffold. Subsequently, a gradual release occurred over the 12 weeks period, reaching (72.56 ± 5.61)% by the end. The scaffold exhibited gradual degradation in the initial 5 weeks, with weight losses of (23.92 ± 3.53)% at week 5, (60.41 ± 2.99)% at week 10, and (65.83 ± 4.2)% at week 12.

### 3.4 Biocompatibility assessment

**Fig. 6:**
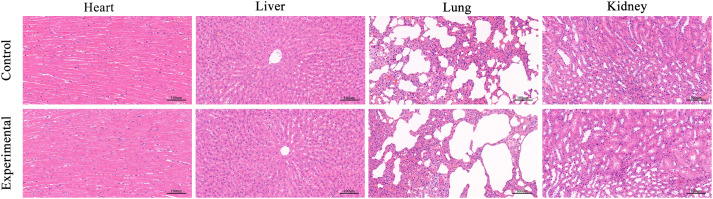
After 12 weeks, the HE staining results of the hearts, livers, lungs and kidneys of the experimental group and the control group were obtained.

The hearts, livers, lungs, and kidneys from both the experimental and control groups were fixed and processed into paraffin sections for HE staining (Fig. 6). Examination of the HE-stained sections revealed an absence of significant pathological changes in the heart, liver, lung, and kidney tissues of the experimental group when compared to the control group. This collective evidence indicates that the scaffold material exhibits commendable biocompatibility, demonstrating minimal local and systemic toxicity in the organism.

### 3.5 Imaging findings of scaffold osteogenesis

**Fig. 7:**
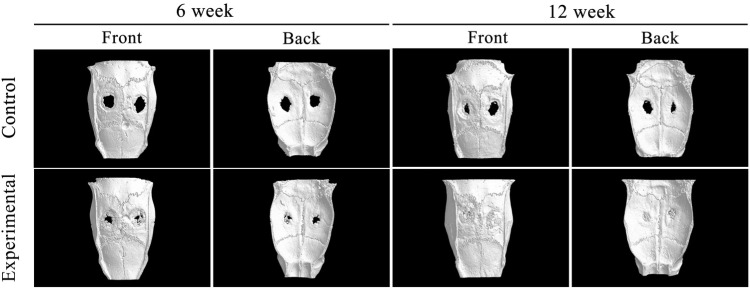
The experimental and control groups were scanned by Micro-CT with three-dimensional imaging after 6 and 12 weeks.

**Fig. 8:**
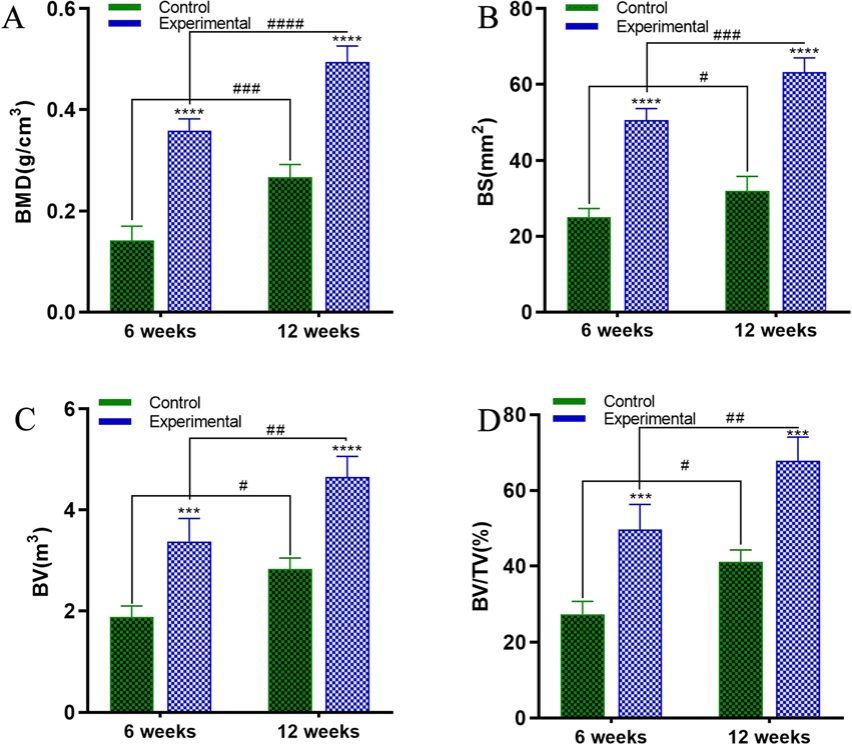
The skull Micro-CT data of the experimental and control groups were subjected to statistical analysis, including (A): The BMD of the two groups of skulls; (B): the BS of the two groups of skulls; (C): the BV of the two groups of skulls; (D): the BV/TV of the two groups of skulls.

The skulls of mice were scanned using Micro-CT and then three-dimensional reconstructions were carried out (Fig. 7). The reconstructed image distinctly delineated the circular boundary of the defect area, revealing significantly enhanced osteogenic activity in the scaffold group compared to the control group. Analysis at the 6th week indicated limited osteogenesis along the margin of the skull defect in the control group, whereas the experimental group exhibited osteogenesis in both the margin and middle of the defect. By Week 12, the experimental group displayed substantial new bone formation covering nearly the entire defect, with minimal porosity. In contrast, the control group exhibited some osteogenesis along the edge but retained a considerable defect area in the middle. Defect area analysis, utilizing each defect center as the origin and a 5 mm diameter, involved measuring the volume of new bone tissue, bone mineral density (BMD), bone surface area (BS), bone volume (BV), and bone volume fraction (BV/TV). Data analysis revealed significantly higher values for BMD, BS, BV, and BV/TV in the experimental group compared to the control group. Statistical analysis corroborated the significant differences between the experimental and control groups (Fig. 8).

## 4 Discussion

Various biomaterials, including PMMA, ceramics, demineralized bone matrix, and calcium phosphate, have been extensively studied for clinical application in hard tissue reconstruction. These materials have shown potential in promoting new bone formation and facilitating the fusion of transplanted bone.^11–14^ However, there remains a paucity of biodegradable materials specifically designed for addressing infectious bone defects. Zhang *et al.*^15^ prepared a collagen/β-tricalcium phosphate scaffold for local drug delivery; Gao *et al.*^16^ fabricated a 3D-printed polyelectrolyte scaffold for the repair of bone defects and the local delivery of SAB. Nabizadeh *et al.*^17^ prepared a nano chitosan/hyaluronic acid hydrogel, which was used to locally inhibit inflammation and support cartilage regeneration; however, these scaffolds still have drawbacks such as poor osteogenic properties or rapid degradation. Thus, the development of a biodegradable material with concurrent osteogenic, biocompatible, and antibacterial properties assumes significant importance. Given numerous studies have shown that HA possesses remarkable biological activity, it holds great potential in osteogenesis and angiogenesis.^18–20^ This study aims to construct a collagen scaffold carrying isoniazid through biomimetic mineralization technology, and to investigate its properties in terms of drug delivery, degradation, biocompatibility and bone formation.

A collagen scaffold loaded with isoniazid was prepared based on the principle of biomimetic mineralization assembly. Phase analysis *via* XRD confirmed that the primary inorganic component of the scaffolds was HA, with no observed phase change during scaffold preparation. Research has shown that the optimal pore size range for bone tissue growth is 200 to 350 micrometers.^21,22^ We analyzed the scaffold using ESEM, and it was observed that the scaffold has a loose and porous structure. This loose and porous structure is conducive to the growth of the tissue. Biodegradability, a key factor in bone tissue engineering scaffolds, demands materials with mechanical properties akin to host tissues and controlled, time-dependent *in vivo* degradation. Ideal scaffolds should provide room for new bone tissue growth. Previously, some scholars had prepared other types of drug-loaded stents. Chen *et al.*^23^ prepared a composite nanofiber, with a encapsulation rate of 47.3 ± 3.4%. Oliva *et al.*^24^ developed a type of RGD-tagged polymeric micellar nanoassemblies, with a drug encapsulation rate of 35%. The drug encapsulation rate of the isoniazid scaffold we obtained was 54 ± 2.34%, which is superior to that of similar types. Sustained drug release in the body ensures prolonged local efficacy. The scaffolds were implanted into mice, and after a specific period of time, they were removed and the remaining drug amounts were measured. This proved that the drug-loaded scaffolds could release drugs slowly and continuously in the body for a long time.

Infected bone defects frequently experience relapses of local infections, impeding the healing process. Utilizing drug-loaded scaffold materials for anti-infection treatment, delivering sustained-release drugs locally, offers a solution to prevent postoperative recurrence and facilitate new bone formation. To assess the scaffold's anti-tuberculosis effect, it was co-cultured with *Mycobacterium tuberculosis*. Detection through the BACTEC MIGT 960 system, anti-“O” staining, and AFS categorized three groups: the experimental group (drug-loaded scaffold and *Mycobacterium tuberculosis* co-culture), the control group (blank scaffold and *Mycobacterium tuberculosis* co-culture), and the blank group (no material added). When the *Mycobacterium tuberculosis* concentration reached a certain level in the MGIT culture tube, the BACTEC MIGT 960 system automatically reported positive. The average reporting time for the Blank Group was 9.11 ± 1.36 days, and for the control group, it was 12.83 ± 2.9 days, with no positive reaction reported in the experimental group after 8 weeks of continuous culture. AFS of the culture medium at 8 weeks revealed *Mycobacterium tuberculosis* in the control and blank groups, but minimal presence in the experimental group, indicating the effective anti-tuberculosis performance of the new isoniazid-loaded scaffold.

Biomaterials, considered foreign entities in the host, may elicit responses or rejections *in vivo*. Optimal biomaterials necessitate superior biocompatibility, ensuring host acceptance without adverse effects on living organs and tissues.^25,26^ Effective bone repair materials should exhibit bone inductivity and promote new bone formation. The biocompatibility and osteogenic effects of scaffolds were explored in a rat skull defect model, employing a 5 mm diameter bone defect model, as smaller bone defects exhibit a strong tendency for self-healing.^10,27^ Two 5 mm-diameter round holes were drilled into rat skulls, with scaffolds implanted in the experimental group and no material in the control group. Biocompatibility and osteogenic effects were assessed at 6 and 12 weeks postoperatively through imaging. Micro-CT three-dimensional reconstruction of the rat skull revealed substantial bone formation in the experimental group's defect center, whereas the control group exhibited limited osteogenesis at the defect's edge. Analyzing parameters (BMD, BS, BV, and BV/TV) showed a significant difference between the experimental and control groups. At 12 weeks postoperatively, HE staining of the heart, liver, lungs, and kidneys indicated robust biocompatibility of the scaffolds.

## 5 Conclusion

We developed an isoniazid-loaded biomimetic mineralized bone collagen scaffold with favorable *in vivo* drug release and degradation properties. *In vitro* cocultivation with *Mycobacterium tuberculosis* confirmed robust anti-tuberculosis efficacy. Upon implantation into rat skulls, the scaffolds demonstrated potent osteoinductive activity, fostering new bone formation. The scaffold's honeycomb structure serves as an effective bridge and medium for bone tissue growth. This biomimetic mineralized collagen scaffold holds promising prospects for treating infectious bone defects. However, it might be necessary to conduct further evaluations of the material's properties from multiple perspectives, such as assessing its osteogenic characteristics at the cellular level.

## Ethical statement

All animal procedures were performed in accordance with the Guidelines for Care and Use of Laboratory Animals of China Three Gorges University and approved by the Animal Ethics Committee of China Three Gorges University. Animal ethics code number:2023660A.

## Ethics approval and consent to participate

In this study, all the research experiments conducted with animals were approved by the Ethical Committee and the responsible authorities of our research organization(s) following all guidelines, regulations, legal, and ethical standards as required for animals.

## Author contributions

All authors contributed to the study conception and design. Material preparation, data collection and analysis were performed by Qian Wang, Xu Fang and Aihua Feng. The first draft of the manuscript was written by Jun-feng Dong and all authors commented on previous versions of the manuscript. All authors read and approved the final manuscript.

## Conflicts of interest

All authors certify that they have no affiliations with or involvement in any organization or entity with any financial interest or non-financial interest in the subject matter or materials discussed in this manuscript.

## Data Availability

We confirm that all data generated or analyzed during this study are included in this published article. Other detailed data for this article are available at Science Data Bank at [https://www.scidb.cn/en/anonymous/WnJlWTdi].
